# Prospective randomized study evaluating the usefulness of a surgical smoke evacuation system in operating rooms for breast surgery

**DOI:** 10.1186/s12995-020-00259-y

**Published:** 2020-05-25

**Authors:** Yutaka Tokuda, Takuho Okamura, Miki Maruta, Mutsuko Orita, Miyuki Noguchi, Toshiyasu Suzuki, Hideaki Matsuki

**Affiliations:** 1 Department of Breast and Endocrine Surgery, Tokai University School of Medicine, Isehara, Japan; 2 Breast Center, Seirei Yokohama Hospital, Yokohama, Japan; 3 Tokai University Hospital Department of Nursing, Isehara, Japan; 4 Department of Materials and Life Sciences, Seikei University, Musashino, Japan; 5 Department of Anesthesiology, Tokai University School of Medicine, Isehara, Japan; 6 Department of Nursing, Tokai University School of Medicine, Isehara, Japan

**Keywords:** Prospective randomized study, Surgical smoke, Evacuation system

## Abstract

**Background:**

No prospective evaluation of surgical smoke evacuation systems has yet been conducted anywhere in the world. A prospective randomized study was conducted to clarify the usefulness of a surgical smoke evacuation system in terms of reducing the quantity of environmental pollutants found in operating room air and reducing the occupational exposure of doctors and nurses involved in surgical procedures to surgical smoke, volatile organic compounds, formaldehyde, etc.

**Methods:**

Operating room environment conditions with and without the use of a surgical smoke evacuation system were measured, and the personal exposure levels of doctors and nurses involved in surgical procedures were also surveyed. Use of the evacuation system was determined randomly, and the procedures involved were breast-conserving surgery and mastectomy, which were treated as stratification factors.

**Results:**

The average total volatile organic compound concentration in the operating room was significantly lower when the evacuation system was used compared with when it was not used. The findings were similar for formaldehyde concentration. Multiple regression analysis for healthcare professionals’ personal exposure levels showed that the evacuation system was a factor that significantly impacted their formaldehyde and acetaldehyde personal exposure levels, which were greatly reduced by the use of the system.

**Conclusion:**

This study’s findings demonstrate the effectiveness of the evacuation systems, which should increase awareness that their benefits take priority over the drawbacks.

**Trial registration:**

The study was conducted after explaining to participants that it was a study of operating room environments in which their participation was voluntary and obtaining their consent. The study was also approved by the Tokai University Hospital clinical research review committee (no. 5R-022) and registered with the UMIN registry (UMIN000029092) on 13, September, 2017- retrospectively registered.

## Introduction

The air environment in operating rooms contains many chemical substances, including anesthetic gas, volatile medical agents used for sterilization and other purposes, and surgical smoke. Since the 1960s, there have been concerns about the harmful effects of these chemical substances on healthcare professionals’ health, and extensive research has been conducted [1–3]. In America, the National Institute for Occupational Safety and Health (NIOSH) [3] recommends the following as environmental exposure standards: 25 ppm or less for nitrous oxide (N_2_O) and 2 ppm or less for volatile anesthetic agent when used alone or 0.5 ppm or less if used in combination with N_2_O. It is therefore mandatory for all anesthetic apparatus to be equipped with a waste anesthetic gas scavenging system. As a result of this equipment, operating room environments are currently able to meet the above standards, for the most part.

Installation of waste anesthetic gas scavenging systems is also now mandatory in Japan, and ventilation-related standards have also become more stringent in terms of safety, which has enabled a reduction in anesthetic gas leakage in operating rooms. However, national standards have not yet been established for reducing exposure to volatile medical agents, chemical substances, and surgical smoke [4–6], nor has an official reporting system on operating room environments been established. An experimental study revealed local exhaust ventilation can significantly reduce airborne particles and volatile organic compounds (VOC) [7]. Thus, a formal and a prospective investigation of personal exposure levels of airborne surgical smoke in actual surgeries is necessary.

Accordingly, for this paper, a prospective randomized study was conducted to clarify the usefulness of a surgical smoke evacuation system in terms of reducing the quantity of environmental pollutants found in operating room air and reducing the occupational exposure of doctors and nurses involved in surgical procedures to surgical smoke, VOC, formaldehyde, etc.

## Methods

Study Methodology: Operating room environment conditions at Tokai University Hospital with and without the use of a surgical smoke evacuation system were measured, and the personal exposure levels of doctors (9 surgeons) and central operating room nurses involved in surgical procedures were also surveyed. Use of the surgical smoke evacuation system was determined randomly, and the procedures involved were breast-conserving surgery and mastectomy for breast cancer patients, which were treated as stratification factors.

### Surgeries and operating rooms for which environment surveys were conducted

Operating room environment surveys were conducted from June 30, 2015, until July 15, 2016, with environmental measurement and personal exposure level surveys conducted for 32 procedures where a surgical smoke evacuation system was used and 30 procedures where it was not used. Of the 18 operating rooms from No. 1 to 18, measurement was carried out for the 12 rooms: The size, ventilation conditions, etc., for each operating room are shown in Table 1. Surgical smoke was allowed to dissipate using the standard room ventilation for cases where the evacuation system was not used.

**Table 1:** Operating Room Size, Ventilation Conditions, Etc

Operating Room No.	Room Area (m^2^)	Room Volume (m^3^)	HEPA Air Flow (m^3^/hr)	Air Exchange Rate (times/hr)	Fresh Air Volume (m^3^/hr)
1	48.3	144.9	4,680	32.3	250
3	63.4	190.2	5,760	30.2	1,000
5	47.5	142.5	4,680	32.8	750
6	53.0	159.0	4,4680	30.0	750
7	62.0	186.0	5,760	30.1	1,000
11	48.3	144.9	4,680	32.2	750
12	48.0	144.0	4,680	32.5	750
13	47.9	143.7	4,680	32.5	750
14A	75.9	227.7	8,160	35.8	1,200
15	47.6	142.8	4,680	32.7	750
17	40.7	112.1	4,680	38.3	700
18	40.9	122.7	4,680	38.1	550

During environmental surveys were conducted, for procedures where the evacuation system was used, the volatile anesthetic used was sevoflurane (SEV) in 11 cases and desflurane (DES) in 21 cases; for procedures where the evacuation system was not used, the anesthetic was SEV in 12 cases and DES in 18 cases.

### Surgical smoke evacuation system: ConMed Aer Defence (Fig. 1)

ConMed Aer Defence, manufactured by Japan Medicalnext Co., Ltd. (Tokyo, Japan), was used as the surgical smoke evacuation system. Aer Defence is designed to reduce and eliminate surgical smoke, aerosols, and odors during surgical procedures. It incorporates a motor with which a high aspiration amount (25 maximum SCFM) may be set. This motor carries surgical smoke aspirated from the surgical site to a filter via a suction tube for filtration treatment. The filter has a three-stage structure. The first filter stage functions as a pre-filter that eliminates large particles and liquid components. The second filter stage, which uses high-performance activated carbon, eliminates and absorbs odors and toxic gas. The third filter stage is a ULPA (Ultra-Low Penetration Air) filter that captures fine particles and micro-organisms of up to 0.10 μm with a 99.9995% efficiency rate.

**Fig. 1:**
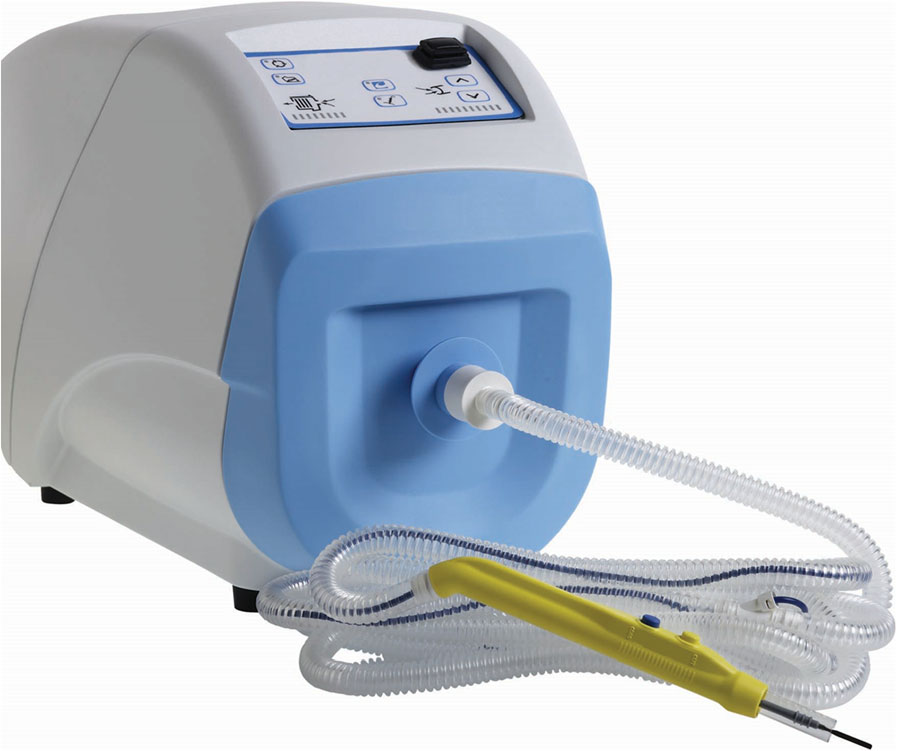
Surgical smoke evacuation system: ConMed Aer Defense

The machine, suction tubes, and other parts were loaned by Japan Medicalnext for the duration of the study.

### Environmental measurement in the operating room

#### Measurement of TVOC (Total volatile organic compounds)

TVOC was measured using a Figaro Engineering Inc. (Osaka, Japan) FTVR-01 TVOC monitor. After measurement was performed on a given survey date, the TVOC data was transferred to a computer using an RS-232C cable. Measurement was carried out from the start to the end of the procedure, with the measurement interval set as 1 min.

#### Formaldehyde measurement

Formaldehyde was measured using a Shinyei Co., Ltd. (Hyogo, Japan) FMM-MD formaldehyde multi-monitor. During measurement, data collected in the measurement device’s internal data logger was transferred to a computer using a USB cable every 30 min. Measurement was performed from the start of the procedure until the end.

#### Dust measurement

Dust was measured using a Shibata Scientific Technology Ltd. (Saitama, Japan) LD-3B-type digital dust meter. This light-scattering measurement device uses a laser diode as an optical source. Data was collected in the device using a logging function at 1-min measurement intervals, then transferred to a computer. Measurement was performed from the start of the procedure until the end.

#### Temperature and humidity measurement

Operating room temperature and humidity were measured using a T&D Corporation (Nagano, Japan) Illuminance-UV Recorder TR-74Ui. The temperature and humidity were measured at 1-min intervals from the start of the procedure until the end.

#### Measurement device installation locations

Devices that performed measurement over time, such as TVOC meters, formaldehyde meters, digital dust meters, and thermo-hygrometers, were installed in two locations: 1.5 m from the operative field (near the operating table), and near the exhaust vent, 3.5 m from the operative field (away from the operating table). The instruments’ variation and calibration were checked before the study.

### Measurement of chemical substance personal exposure levels

Surgeons, surgical assistants, direct care nurses (scrub nurses), indirect care nurses (circulating nurses), and anesthetists were asked to wear a Sigma-Aldrich Japan (Tokyo, Japan) sampler (DSD-DNPH sampler: Diffusive Sampler for Determination with 2, 4-dinitrophenylhydrazine; length: 5 cm; width: 8 mm; weight: 5 g) on sterile clothing on their chest during surgery. Once exposure was over, the sampler was sealed, and extraction of chemical substances from the DSD-DNPH sampler was performed by eluting an aldehyde/ketone-DNPH derivative with 5.0 mL of acetonitrile. Analysis was performed using a Hitachi High-Technologies (Tokyo, Japan) mass spectrograph (GC-MS/MS TSQ Quantum XLS Ultra) analytical instrument, which measured the personal exposure concentration of organic compounds (formaldehyde, acetone, acetaldehyde) [8].

### Statistical analysis method

#### Setting the number of cases

Two groups (α = 0.05, 1-β = 0.8) were compared, with 30 cases per group.

#### Analysis method

Using the obtained data, basic statistics such as average values and standard deviations were calculated. The average values were compared for each group, and a Student’s t-test or Welch’s t-test was conducted for unpaired t-tests, while correlation was tested by calculating the Pearson’s correlation coefficient. Multiple regression analysis was also performed. The following statistical software was used: SPSS v21.0 (serial no. 16071376) and HALWIN v7 (serial no. 111908233).

## Results

### Total volatile organic compound concentration in operating room

**Fig. 2:**
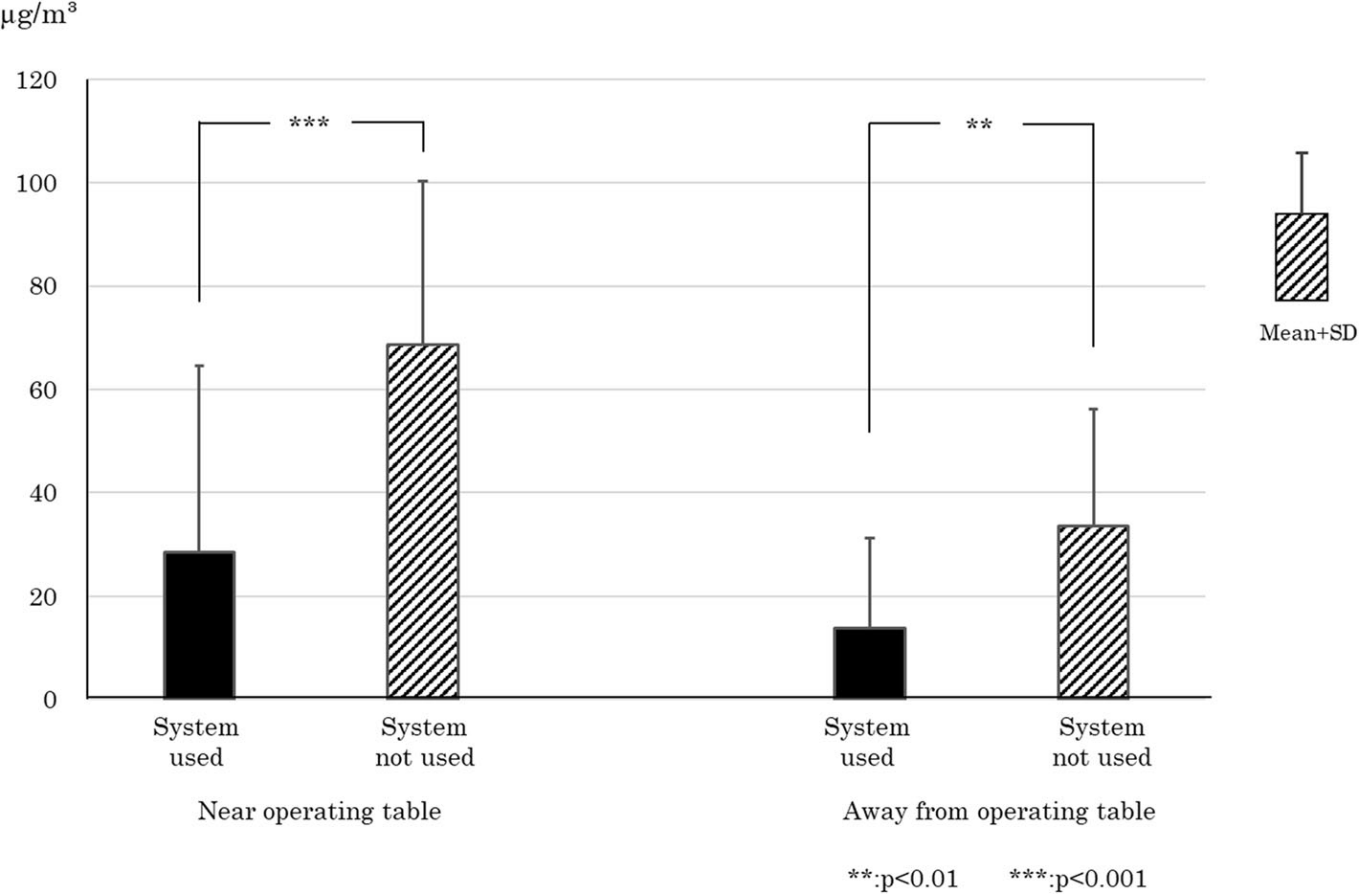
Effects of surgical smoke evacuation system on TVOC. The evacuation system significantly reduced the TVOC concentration in the operating room

The TVOC concentration at the two locations (“near the operating table” and “away from the operating table”) in 31 cases where the evacuation system was used and 30 cases where it was not used is shown in Fig. 2. One case among the evacuation system group was excluded from analysis for the trouble of the measurement machine system. The average TVOC concentration ± standard deviation near the operating table when using the system was 28.3 ± 36.16 μg/m^3^, while the average concentration when not using the system was 68.5 ± 31.6 μg/m^3^. The average concentration when using the system was significantly lower than when not using the system (*p*<0.001). Away from the operating table, the average concentration was 13.8 ± 17.4 g/m^3^ when using the system and 33.6 ± 21.5 g/m^3^ when not using it. The average concentration was again significantly lower when the system was used compared with when it was not used (*p* < 0.01).

**Fig. 3:**
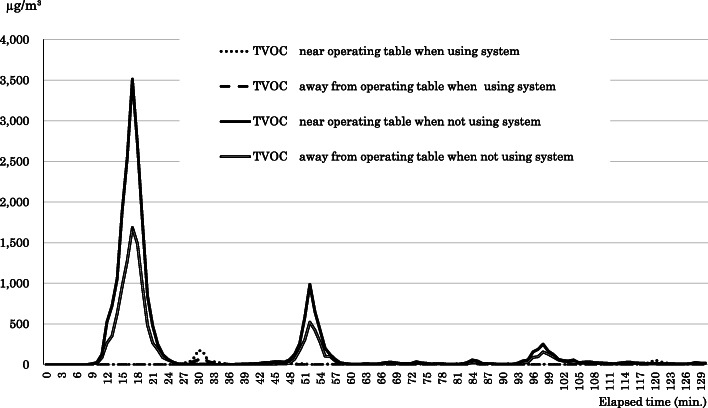
The TVOC concentration changes with time in one of the operating rooms. The TVOC concentration changes with time in one of the operating rooms with or without the evacuation system

As an example, Fig. 3 shows changes in TVOC concentration over time for a procedure performed in operating room No. 12 with the evacuation system and another procedure performed without using the system. There were three significant TVOC concentration peaks during the procedure without the evacuation system, exceeding 3000 μg/m^3^ near the operating table. Conversely, during the procedure where the evacuation system was used, the level peaked at around 175 μg/m^3^ 30 min after the operation started.

### Dust concentration in operating room

The average dust concentration values and standard deviations at the two locations (“near the operating table” and “away from the operating table”) when the evacuation system was used and when it was not used were measured. The average dust concentration ± standard deviation near the operating table when using the system was 4.3 ± 5.2 μg/m^3^, and when not using it, the average concentration was 4.8 ± 6.9 μg/m^3^. Away from the operating table, the respective figures were 3.6 ± 3.5 μg/m^3^ and 3.5 ± 3.4 μg/m^3^. No statistically significant differences between the two groups were observed. These figures are extremely low when compared with Japan’s indoor environment standard of 0.15 mg/m^3^ or less (150 μg/m^3^), as stipulated in the Act on Maintenance of Sanitation in Buildings.

### Formaldehyde concentration in operating room

**Fig. 4:**
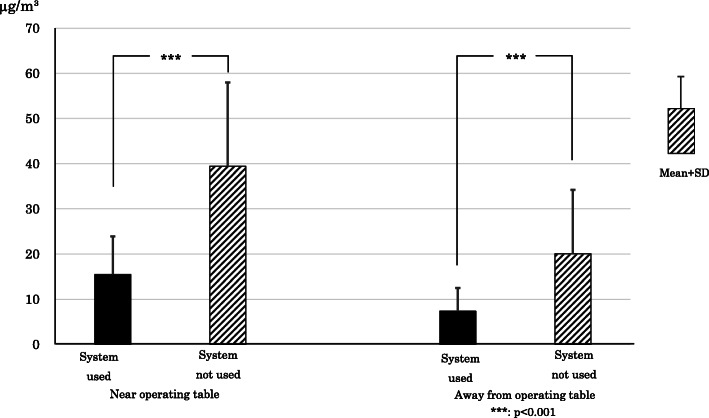
Effects of surgical smoke evacuation system on formaldehyde concentration. The evacuation system significantly reduced formaldehyde concentration in the operating room

The average formaldehyde concentration values and standard deviations at the two locations (“near the operating table” and “away from the operating table”) when the evacuation system was used and when it was not used are shown in Fig. 4. The average formaldehyde concentration ± standard deviation near the operating table when using the system was 15.5 ± 8.4 μg/m^3^, and when not using it, the average concentration was 39.4 ± 18. 6 μg/m^3^. The average concentration when using the system was significantly lower than when not using the system (*p*<0.001). Away from the operating table, the average formaldehyde concentration value was 7.3 ± 5.1 μg/m^3^ when using the system and 20.1 ± 14.1 μg/m^3^ when not using it, so the average concentration was likewise significantly lower when the system was used versus when it was not used (*p* < 0.001).

### Temperature and humidity in operating room

The temperature near the operating table and away from the operating table when the evacuation system was used and when it was not used was maintained at 24 to 25 °C, and no significant variation was observed.

The humidity near the operating table and away from the operating table when the evacuation system was used and when it was not used was maintained at 43.5 to 48.2%, and as was the case for temperature, no humidity differences were observed.

### Formaldehyde personal exposure level measurement results

**Fig. 5:**
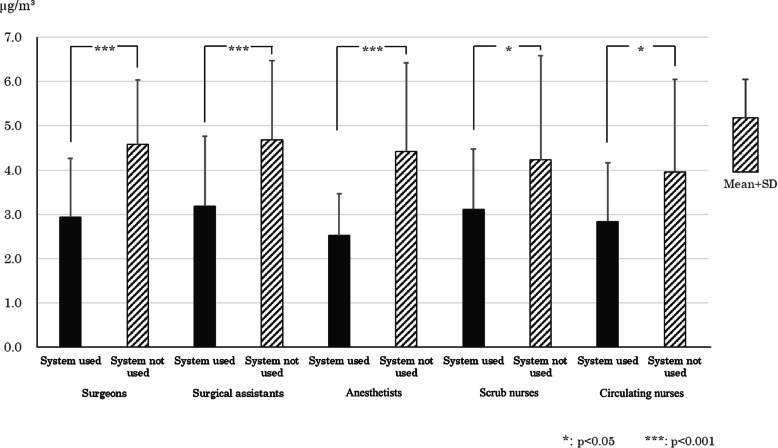
Quantity of personal exposure to formaldehyde with or without the evacuation systems

The average formaldehyde exposure concentration ± standard deviation for surgeons, surgical assistants, anesthetists, direct care nurses (scrub nurses), and indirect care nurses (circulating nurses), broken down according to whether the evacuation system was used or not, is shown in Fig. 5. For surgeons, surgical assistants, anesthetists, scrub nurses, and circulating nurses alike, the value was significantly lower for the “system used” group compared with the “system not used” group (*p* < 0.05 or *p* < 0.001). However, for both groups and all healthcare professional categories, the maximum formaldehyde exposure concentration did not exceed 100 μ/m^3^ (80 ppb), which is the indoor concentration limit suggested by Japan’s Ministry of Health, Labor and Welfare.

### Acetaldehyde personal exposure level measurement results

**Fig. 6:**
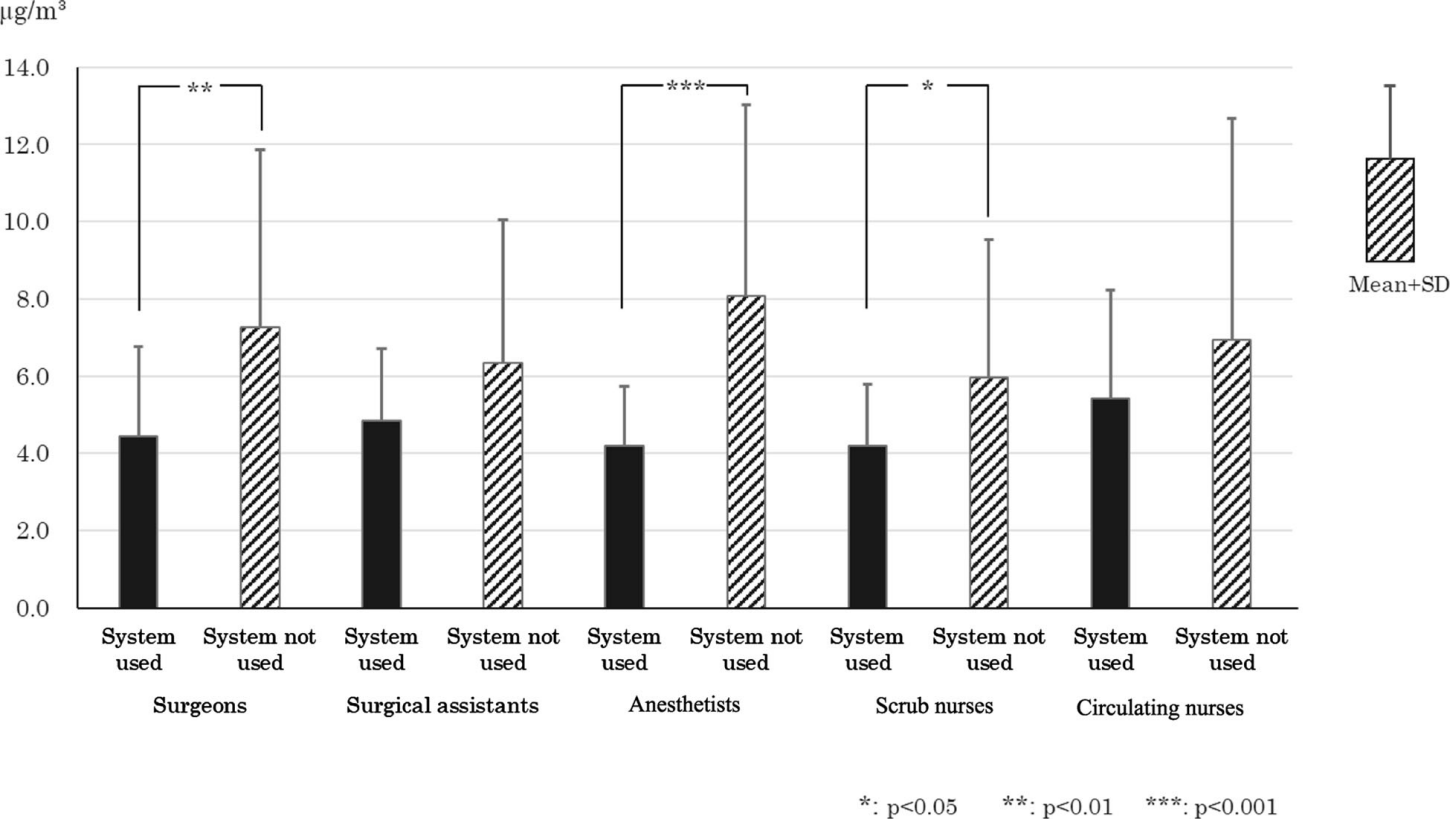
Quantity of personal exposure to acetaldehyde with or without the evacuation systems

The average acetaldehyde exposure concentration ± standard deviation for surgeons, surgical assistants, anesthetists, direct care nurses (scrub nurses), and indirect care nurses (circulating nurses), broken down according to whether the evacuation system was used or not, is shown in Fig. 6. The healthcare professionals for whom the results were significantly lower when the evacuation system was used versus when it was not used were surgeons, anesthetists, and scrub nurses (*p* < 0.05, *p* < 0.01, or *p* < 0.001).

### Acetone personal exposure level measurement results

**Fig. 7:**
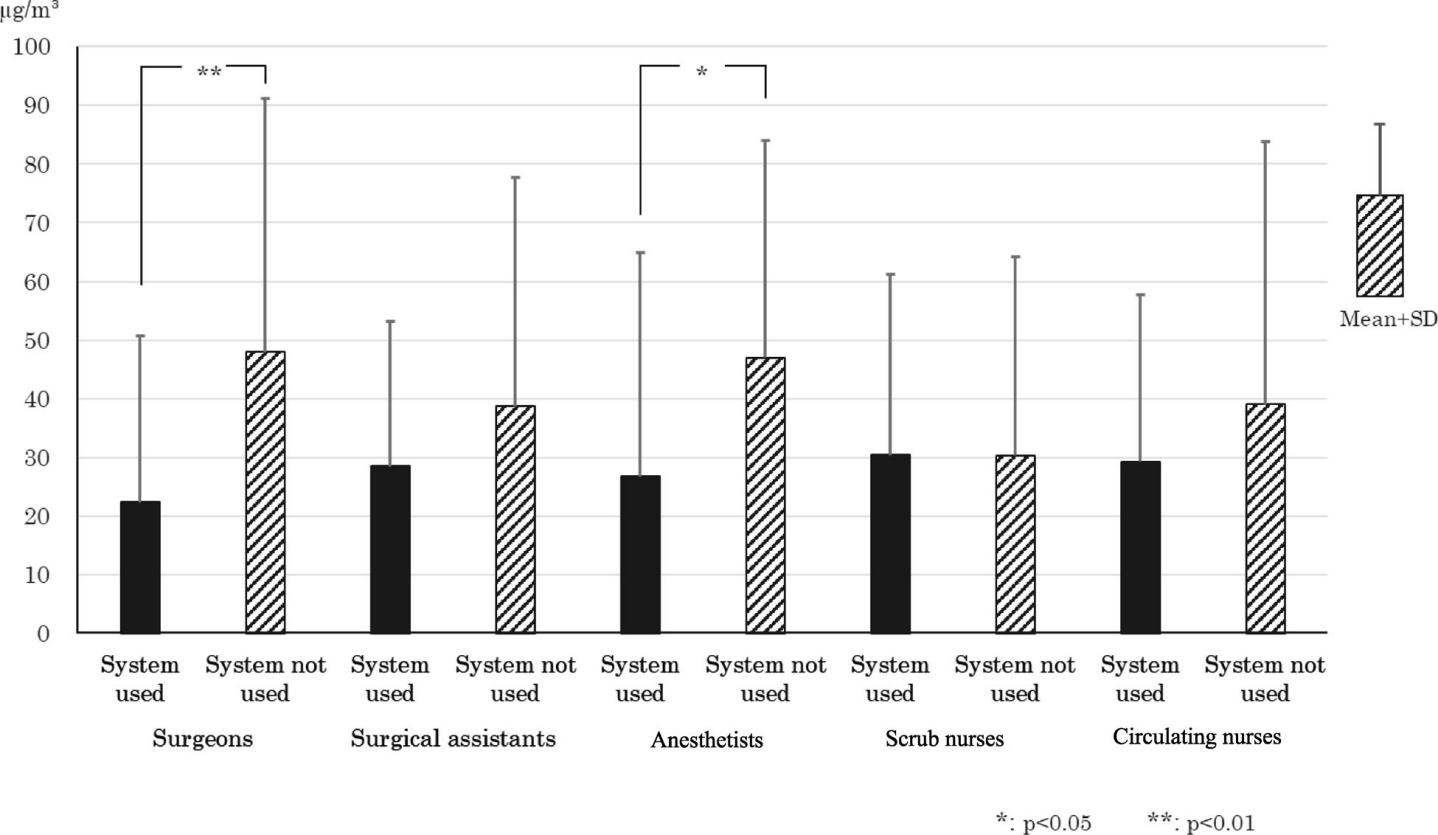
Quantity of personal exposure to acetone with or without the evacuation systems

The average acetone exposure concentration ± standard deviation for surgeons, surgical assistants, anesthetists, direct care nurses (scrub nurses), and indirect care nurses (circulating nurses), broken down according to whether the evacuation system was used or not, is shown in Fig. 7. The healthcare professionals for whom the results were significantly lower when the evacuation system was used versus when it was not used were surgeons and anesthetists (*p* < 0.05 or *p* < 0.01).

### Multiple regression analysis for healthcare professionals’ personal exposure levels

To test the relationship between the formaldehyde, acetaldehyde, and acetone personal exposure levels measured for healthcare professionals and various factors, a multiple regression analysis (stepwise method) was performed. The following were treated as independent variables: years of experience as a surgeon, whether or not the evacuation system was used, the type of mastectomy procedure, the type of anesthetic (sevoflurane: 0, desflurane: 1), operating time (minutes), operating room area (m^2^), operating room volume (m^3^), HEPA air flow (passage through HEPA filter: m^3^/hr), air exchange rate (per hour), and fresh air volume (fresh air intake volume: m^3^). The dependent variables were the formaldehyde, acetaldehyde, and acetone personal exposure levels of surgeons, surgical assistants, anesthetists, scrub nurses, and circulating nurses.

The formaldehyde personal exposure level multiple regression analysis results are shown in Table 2. For the decision variable (adjusted R^2^), the results were: 0.253 for surgeons, 0.158 for surgical assistants, 0.329 for anesthetists, 0.066 for scrub nurses, and 0.144 for circulating nurses. With regard to stepwise method independent variables, the factor remaining at the end for all healthcare professionals was use of the evacuation system. The acetaldehyde personal exposure level multiple regression analysis results are shown in Table 2. For the decision variable (adjusted R^2^), the result was highest for anesthetists (0.257), while the factor remaining at the end was use of the evacuation system for surgeons, surgical assistants, anesthetists, and scrub nurses.

**Table 2:** Multiple Regression Analysis

**Results – Formaldehyde**		
Surgeons	β (coefficient)	*p*-value
Constant	4.578	0.000
Evacuation system used	-1.647	0.000
Decision variable (adjusted R2)	0.253	
Surgical assistants	β (coefficient)	*p*-value
Constant	4.684	0.000
Evacuation system used	-1.506	0.001
Decision variable (adjusted R2)	0.158	
Anesthetists	β (coefficient)	*p*-value
Constant	4.899	0.000
Evacuation system used	-1.932	0.000
Total mastectomy	-0.952	0.014
Decision variable (adjusted R2)	0.329	
Scrub nurses	β (coefficient)	*p*-value
Constant	4.228	0.000
Evacuation system used	-1.117	0.025
Decision variable (adjusted R2)	0.066	
Circulating nurses	β (coefficient)	*p*-value
Constant	3.353	0.000
Evacuation system used	-1.182	0.007
Anesthetic	1.013	0.025
Decision variable (adjusted R2)	0.144	
**Results - Acetaldehyde**		
Surgeons	β (coefficient)	*p*-value
Constant	12.439	0.000
Evacuation system used	-2.69	0.003
Operating time (min.)	-0.039	0.009
Surgeon’s years of experience	-0.086	0.043
Decision variable (adjusted R2)	0.218	
Surgical assistants	β (coefficient)	*p*-value
Constant	12.338	0.000
Fresh air volume	-0.005	0.010
Operating time (min.)	-0.024	0.034
Evacuation system used	-1.376	0.049
Decision variable (adjusted R2)	0.198	
Anesthetists	β (coefficient)	*p*-value
Constant	6.915	0.000
Evacuation system used	-3.99	0.000
Anesthetic	1.921	0.014
Decision variable (adjusted R2)	0.257	
Scrub nurses	β (coefficient)	*p*-value
Constant	5.957	0.000
Evacuation system used	-1.765	0.014
Decision variable (adjusted R2)	0.082	
Circulating nurses	β (coefficient)	*p*-value
Constant	10.084	0.000
Operating time (min.)	-0.041	0.024
Decision variable (adjusted R2)	0.067	

Based on the above results, it is clear that the evacuation system was a factor that significantly impacted healthcare professionals’ formaldehyde and acetaldehyde personal exposure levels, which were greatly reduced by the use of the system.

## Discussion

This study prospectively evaluated the usefulness of a movable surgical smoke evacuation system in the operating room. To date, this issue has not been reported on either in Japan or other countries. The concentrations of TVOC, dust, and formaldehyde in the operating room were measured, along with the personal exposure levels of healthcare professionals to volatile organic compounds such as formaldehyde, acetaldehyde, and acetone during a procedure.

The results showed that the average total volatile organic compound (TVOC) concentration in the operating room was significantly lower when the evacuation system was used compared with when it was not used, whether at a position near the operating table or away from the operating table. The findings were similar for formaldehyde concentration in the operating room.

The evacuation system used in this study houses three filters. The first filter stage is a pre-filter that eliminates large particles and liquid components, while the second filter stage, which uses high-performance activated carbon, eliminates and absorbs odors and toxic gas. The third filter stage is a ULPA (Ultra-Low Penetration Air) filter that captures fine particles and micro-organisms of up to 0.10 μm with a 99.9995% efficiency rate. The results of this experiment suggest that the three filters were effective.

In terms of healthcare professionals’ formaldehyde personal exposure levels, for surgeons, surgical assistants, anesthetists, scrub nurses, and circulating nurses alike, the results were significantly lower for the group for which the evacuation system was used compared with the group for which it was not used. This meant surgical smoke contained formaldehyde as previously identified [4–6]. Furthermore, processing the placement of tissue specimens into formaldehyde should be legally done in a special room in Japan. For surgeons, anesthetists, and scrub nurses, acetaldehyde personal exposure levels were significantly lower for the group for which the evacuation system was used compared with the group for which it was not used. And with regard to acetone, for surgeons and anesthetists, the levels were significantly lower for the evacuation system group compared with the group for which it was not used. These results again indicate the usefulness of the evacuation system.

Healthcare professionals in operating rooms are exposed to surgical smoke and volatile organic compounds in the work environment on a daily basis. In this study, the environmental concentration was quite low when compared with the Ministry of Health, Labour and Welfare’s recommended indoor concentration value of 80 ppb (0.08 ppm), and it was rare for TVOC concentration to exceed the level of 400 μg/m^3^, which is the provisional guideline suggested by the same Ministry in 2001. However, while these concentration levels may be low, the chronic effects on health due to exposure over an extended period of time are a concern. Generally, the most common obstacles to the implementation of surgical smoke control practice are the reduction in maneuverability and generation of noise that accompany the installation of evacuation equipment, which have led to resistance from surgeons [9]. This study’s findings demonstrate the effectiveness of evacuation systems, which should increase awareness that their benefits take priority over the drawbacks. Therefore, rather than focusing on cost effectiveness, surgeons should take the initiative in conducting efforts to minimize exposure to surgical smoke for operating room colleagues and workers, as well as for themselves.

In the future, it would be preferable if domestic as well as global standards were established for reducing exposure to volatile medical agents, chemical substances, and surgical smoke in operating rooms and their concentration levels were monitored during surgical procedures.

It would also be preferable if operating rooms were bacteriologically clean. This study has demonstrated that evacuation systems are effective. Hopefully, whether they are also bacteriologically and virologically effective will also be evaluated in the future, and evacuation system manufacturers will release data on their organic compound elimination rate.

## Conclusion

This study’s findings demonstrate the effectiveness of the evacuation systems, which should increase awareness that their benefits take priority over the drawbacks.

## Data Availability

The data that support the findings of this study are available from the corresponding author upon reasonable request.
